# Evidence for Effective Multiple K^+^-Current Inhibitions by Tolvaptan, a Non-peptide Antagonist of Vasopressin V_2_ Receptor

**DOI:** 10.3389/fphar.2019.00076

**Published:** 2019-02-18

**Authors:** Te-Ling Lu, Wei-Ting Chang, Chee-Hong Chan, Sheng-Nan Wu

**Affiliations:** ^1^School of Pharmacy, China Medical University, Taichung, Taiwan; ^2^Division of Cardiovascular Medicine, Chi-Mei Medical Center, Tainan, Taiwan; ^3^Department of Nephrology, Chang Bing Show Chwan Memorial Hospital, Changhua, Taiwan; ^4^Department of Physiology, National Cheng Kung University Medical College, Tainan, Taiwan; ^5^Institute of Basic Medical Sciences, National Cheng Kung University Medical College, Tainan, Taiwan

**Keywords:** tolvaptan, M-type K^+^ current, delayed-rectifier K^+^ current, *erg*-mediated K^+^ current, membrane potential, pituitary cell, renal epithelial cell

## Abstract

Tolvaptan (TLV), an oral non-peptide antagonist of vasopressin V_2_ receptor, has been increasingly used for managements in patients with hyponatremia and/or syndrome of inappropriate antidiuretic hormone secretion. However, none of the studies have thus far been investigated with regard to its possible perturbations on membrane ion currents in endocrine or neuroendocrine cells. In our electrophysiological study, the whole-cell current recordings showed that the presence of TLV effectively and differentially suppressed the amplitude of delayed rectifier K^+^ (*I*_K(DR)_) and M-type K^+^ current (*I*_K(M)_) in pituitary GH_3_ cells with an IC_50_ value of 6.42 and 1.91 μM, respectively. This compound was also capable of shifting the steady-state activation curve of *I*_K(M)_ to less depolarized potential without any appreciable change in the gating charge of this current. TLV at a concentration greater than 10 μM also suppressed the amplitude of *erg*-mediated K^+^ current or the activity of large-conductance Ca^2+^-activated K^+^ channels; however, this compound failed to alter the amplitude of hyperpolarization-activated cation current in GH_3_ cells. In vasopressin-preincubated GH_3_ cells, TLV-mediated suppression of *I*_K(M)_ remained little altered. Under current-clamp condition, we also observed that addition of TLV increased the firing of spontaneous action potentials in GH_3_ cells and further addition of flupirtine could reverse TLV-mediated elevation of the firing. In Madin-Darby canine kidney (MDCK) cells, the K^+^ current elicited by long ramp pulse was also effectively subject to inhibition by this compound. Findings from the present study were thus stated as saying that the suppression by TLV of multiple type K^+^ currents could be direct and independent of its antagonism of vasopressin V_2_ receptors. Our study also reveals an important aspect that should be considered when assessing aquaretic effect of TLV or its structurally similar compounds.

## Introduction

Tolvaptan (TLV; Samsca® or Jinarc®) is recognized as an oral aquaretic agent that functions as a selective, competitive antagonist of vasopressin V_2_ receptor used to treat hyponatremia associated with congestive heart failure, cirrhosis, or the syndrome of inappropriate antidiuretic hormone (Izumi et al., 2014; Aylwin et al., 2015; Verbalis et al., 2016; Clark et al., 2017; Der-Nigoghossian et al., 2017; Dunlap et al., 2017; Felker et al., 2017; Konstam et al., 2017; Wu et al., 2017a; Berardi et al., 2018; Kogure et al., 2018; Matsukawa et al., 2018; Morris et al., 2018; Oguri et al., 2018a; Sigal et al., 2018; Takimura et al., 2018; Vidic et al., 2019). It was noted to be effective at improving the hyponatremic conditions that may occur in different pathologic conditions including that following pituitary surgery (Izumi et al., 2014; Janneck et al., 2014; Ahluwalia et al., 2015; Aylwin et al., 2015; Ichimura et al., 2015; Gralla et al., 2017; Berardi et al., 2018). Alternatively, a previous study showed the ability of the compounds recognized as the blockers of vasopressin V_1B_ receptors, to produce additional antidepressant and anxiolytic profiles (Iijima et al., 2014). TLV clinically used was also reported to improve cognitive function (Soiza and Talbot, 2011; Graziani et al., 2012; Ahluwalia et al., 2015; Verbalis et al., 2016; Der-Nigoghossian et al., 2017).

The KCNQ2, KCNQ3, and KCNQ5 genes are known to encode the core subunits of K_V_7.2, K_V_7.3, and K_V_7.5 channels, respectively. The increased activity of these K_V_ channels in neuron, or endocrine or neuroendocrine cells can generate a unique population of K^+^ current, namely, the M-type K^+^ current (*I*_K(M)_), which exhibits to possess a slowly activating and deactivating property (Brown and Yu, 2000; Quintero et al., 2005; Shu et al., 2007; Wu et al., 2012a; Hsu et al., 2014; Chen et al., 2018). Targeting *I*_K(M)_ is growingly recognized as an adjunctive regimen for the treatment of many neurological disorders. Alternatively, voltage-gated K^+^ (K_V_) channels play an essential role in determining membrane excitability and the delayed rectifier K^+^ channels are ubiquitous in endocrine cells. A causal relationship between K_V_3 (or KCNC) and the delayed rectifier K^+^ current (*I*_K(DR)_) has been previously established (Rudy and McBain, 2001). The K_V_3 subfamily of K_V_ channels is characterized by its biophysical properties exhibiting to have positively shifted voltage dependency and fast deactivation rate. These properties can be expected to limit the Na^+^ channel, thereby leading to depolarization block and accommodation of repetitive firing at high frequencies (Rudy and McBain, 2001; Tateno and Robinson, 2007). K_V_ channels from the K_V_3.1-K_V_3.2 types are the major determinants of *I*_K(DR)_ in pituitary GH_3_ cells (Wang et al., 2008; So et al., 2017). However, whether TLV, expected to be a non-peptide vasopressin antagonist, exerts any perturbations on these types of K^+^ currents (e.g., *I*_K(M)_ and *I*_K(DR)_) or on membrane potential remains largely unexplored, although a previous report showed the ability of TLV to suppress the store-operated Ca^2+^ entry through an interaction with the Orail1 protein (Rahman and Rahman, 2017) and to increase cytosolic Ca^2+^ in Madin-Darby canine kidney (MDCK) cells (Tamma et al., 2017).

For these considerations described above, we sought to determine whether TLV or other related compounds could produce any perturbations on membrane ion channels in endocrine cells (e.g., pituitary tumor [GH_3_] cells) and in renal tubular cells (e.g., MDCK cells). In particular, Ionic currents studied include *I*_K(M)_, *I*_K(DR)_, *erg*-mediated K^+^ current (*I*_K(erg)_), large-conductance Ca^2+^-activated K^+^ (BK_Ca_) channel, and hyperpolarization-activated cation current (*I*_h_). Changes in membrane potential in the presence of TLV were also investigated under current-clamp condition. Unexpectedly, despite being aquaretic action, this drug at clinically relevant concentrations was capable of suppressing various types of K^+^ channels effectively in GH_3_ and MDCK cells.

## Materials and Methods

### Chemicals and Solution

For the present study, tolvaptan (TLV; Samsca® or Jinarc®, OPC-41061, *N*-[4-[(7-chloro-2,3,4,5-tetrahydro-5-hydroxy-1*H*-1-benzazepin-1-yl)carbonyl]-3-methylphenyl]-2-methylbenzamide,C_26_H_25_ClN_2_O_3_), ivabradine and PD-118057 (2-[[4-[2-(3,4-Dichlorophenyl)ethyl]phenyl]amino]benzoic acid) were obtained from Tocris (Union Biomed Inc., Taipei, Taiwan), flupirtine, linopirdine, [Arg^8^]-vasopressin (vasopressin), nonactin, tetrodotoxin and thyrotropin releasing hormone were from Sigma-Aldrich (St Louis, MO), and pioglitazone was from Takeda Pharmaceuticals (Tokyo, Japan). The culture media, fetal bovine, calf or horse serum, L-glutamine and trypsin/EDTA were obtained from Invitrogen (Carlsbad, CA), unless stated otherwise. All other chemicals were of the highest purity commercially available, and deionized water used throughout the experiments was made from a Milli-Q water purification system (Millipore, Bedford, MA).

The composition of bath solution (i.e., normal Tyrode's solution) used in this study was 136 mM NaCl, 5.4 mM KCl, 1.8 mM CaCl_2_, 0.53 mM MgCl_2_, 5.5 mM glucose, and 5.5 mM HEPES-NaOH buffer, pH 7.4. To measure macroscopic K^+^ currents (e.g., *I*_K(DR)_ or *I*_h_) and to preclude contamination of Cl^−^ currents, we filled the patch pipettes with a solution which contained 130 mM K-aspartate, 20 mM KCl, 1 mM KH_2_PO_4_, 1 mM MgCl_2_, 3 mM Na_2_ATP, 0.1 mM Na_2_GTP, 0.1 mM EGTA, and 5 mM HEPES-KOH buffer, pH 7.2. For measurements of whole-cell *I*_K(M)_ or *I*_K(erg)_, or single BK_Ca_-channel activity, we used a high K^+^-bathing solution consisting of 145 mM KCl, 0.53 mM MgCl_2_, and 5 mM HEPES-KOH buffer, pH 7.4, while the recording pipette used was filled with a solution containing 145 mM KCl, 2 mM MgCl_2_ and 5 mM HEPES-KOH buffer, pH 7.2. The free Ca^2+^ concentration in bath medium was estimated, as the dissociation constant for EGTA and Ca^2+^ (at pH 7.2) was assumed to be 0.1 μM. For example, to provide 0.1 μM Ca^2+^, we added 0.5 mM CaCl_2_ and 1 mM EGTA.

### Cell Preparations

GH_3_, a clonal cell line derived from a rat prolactin-secreting pituitary tumor, was obtained from the Bioresources Collection and Research Center (BCRC-60015; Hsinchu, Taiwan). Cells were routinely cultured in Ham's F-12 medium supplemented with 15% heat-inactivated horse serum (v/v), 2.5% fetal calf serum (v/v), and 2 mM L-glutamine (Liu et al., 2009; So et al., 2017, 2018). In another separate set of experiments, we treated GH_3_ cells with vasopressin (1 μM) at 37°C for 6 h. The MDCK cell line (BCRC-60004), a canine renal tubular cell line, was also obtained from the Bioresource Collection and Research Center (Hsinchu, Taiwan). MDCK cells were maintained and subcultured in Dulbecco's modified Eagle's medium supplemented with 10% fetal bovine serum (v/v) (Jan et al., 1999; Wu et al., 2015). These cells were maintained at 37°C in a humidified environment of 5% CO_2_/95% air. We commonly replaced culture medium every 2 days for removal of non-adhering cells. For long-term storage, we froze the cells in culture media containing 10% dimethyl sulfoxide, and kept in liquid nitrogen. Cell viability was commonly evaluated using a WST-1 cell proliferation assay and an ELISA reader (Dynatech, Chantilly, VA).

### Electrophysiological Measurements

On the day of the measurements, GH_3_ or MDCK cells were dissociated, and an aliquot of cell suspension was transferred to a home-made recording chamber affixed to the stage of a DM-IL inverted microscope (Leica, Wetzlar, Germany). For visualizing change in cell size during the recordings, the microscope was coupled to a digital video system (DCR-TRV30; Sony, Japan) with a magnification of up to 1500×. The cells examined were immersed at room temperature (20–25°C) in normal Tyrode's solution, the composition of which is described above. The recording electrodes were pulled from Kimax-51 capillaries (#34500; Kimble Glass, Vineland, NJ) using either a PP-830 puller (Narishige, Tokyo, Japan) or a P-97 Flaming/Brown micropipette puller (Sutter, Novato, CA), and their tips were fire-polished with an MF-83 microforge (Narishige). The electrodes used during the recordings had a tip resistance of 3–5 MΩ when filled with different internal solution described above. Ion currents were measured in either whole-cell, cell-attached or inside-out configuration of a standard patch-clamp technique with an RK-400 (Bio-Logic, Claix, France) or an Axopatch-200B (Molecular Devices, Sunnyvale, CA) amplifier (So et al., 2017; Wu et al., 2017b). Changes in membrane potential were measured under current-clamp configuration. All potentials were commonly offset for liquid junction potentials which arose at the electrode tip when the composition of the pipette solution was different from that in the bath. Single BK_Ca_-channel activity measured from GH_3_ cells was analyzed using pCLAMP 10.2 (Molecular Devices).

### Data Recordings and Analyses

The signals achieved were examined and analyzed offline using either pCLAMP 10.2 (Molecular Devices), 64-bit OriginPro 2016 (OriginLab, Northampton, MA), or custom-made macros created from Excel 2013 which was run under Windows 10 (Microsoft, Redmond, WA). Current signals were low-pass filtered at 1 or 3 kHz. Through digital-to-analog conversion, the pCLAMP-generated voltage-step profiles with various rectangular or ramp waveforms were computer driven to evaluate either the current vs. voltage (*I-V*) relations or the steady-state activation curve for different types of ionic currents (e.g., *I*_K(M)_) obtained with or without addition of different tested compounds.

To determine percentage inhibition of TLV on *I*_K(M)_ or *I*_K(DR)_, we compared current amplitudes obtained in the presence of different TLV concentrations (0.1–100 μM) with the values from control experiments. To measure *I*_K(M)_, cells were immersed in high-K^+^, Ca^2+^-free solution and, once the whole-cell current recording was achieved, each cell was depolarized from −50 to +10 mV with a duration of 1 s, while to record *I*_K(DR)_, they were bathed in Ca^2+^-free Tyrode's solution and the depolarizing pulse from −50 to +50 mV was delivered. The concentration-dependent relationships of TLV on the inhibition of *I*_K(M)_ or *I*_K(DR)_ amplitude were fitted to a modified Hill function by nonlinear least-squares regression analysis; that is,

$$\text{Percentage inhibition }\left(\%\right)=\frac{{\text{E}}_{\max}\times {\left[\text{C}\right]}^{{\text{n}}_{\text{H}}}}{{\left[\text{C}\right]}^{{\text{n}}_{\text{H}}}{\text{+IC}}_{\text{50}}^{{\text{n}}_{\text{H}}}},$$

where IC_50_ is the concentration required for a 50% inhibition, [C] the TLV concentration applied, n_H_ the Hill coefficient, and E_max_ maximal inhibition of *I*_K(M)_ or *I*_K(DR)_ amplitude produced by TLV.

To evaluate effect of TLV on the steady-state activation curve of *I*_K(M)_ in GH_3_ cells, we held each cell at the level of −50 mV and then applied the voltage pulses from−50 to various test potentials ranging between −50 and +10 mV with a duration of 1 s which were delivered from pCLAMP 10.2 program through digital-to-analog conversion. The activation curve of the current taken with or without addition of TLV (10 μM) was fitted by the Boltzmann function:

$$\frac{\text{I}}{{\text{I}}_{\max}}=\frac{1}{1+\text{exp}\left[-\langle \text{V}-{\text{V}}_{1/2}\rangle \text{qF}/\text{RT}\right]},$$

where I_max_ is the maximal *I*_K(M)_ amplitude, V_1/2_ the voltage at which there is half-maximal activation of the current, q the apparent gating charge (i.e., the charge across the membrane electric field between closed and open conformations), R the universal gas constant, F Faraday's constant, and T the absolute temperature.

### Statistical Analyses

Linear or nonlinear curve-fitting to the experimental data was appropriately implemented by using either pCLAMP 10.2 (Molecular Devices), OriginPro 2016 (OriginLab), or Microsoft Excel 2013 (i.e., “Solver” Add-in). The data are presented as mean value ± standard error of the mean (SEM) with sample sizes (n) indicating the cell number from which the results were taken; error bars are plotted as SEM. Paired or unpaired Student's *t*-tests were initially used for the statistical analyses. However, when the statistical difference among different groups was necessarily evaluated, we further implemented the *post-hoc* Duncan multiple comparisons among them. Statistical analyses were performed using IBM SPPSS version 20.0 (IBM Corp., Armonk, NY). Differences between values were considered significant when *P* < 0.05.

## Results

### Effect of Tolvaptan (TLV) on Delayed-Rectifier K^+^ Current (I_K(DR)_) in GH_3_ Cells

**Figure 1:**
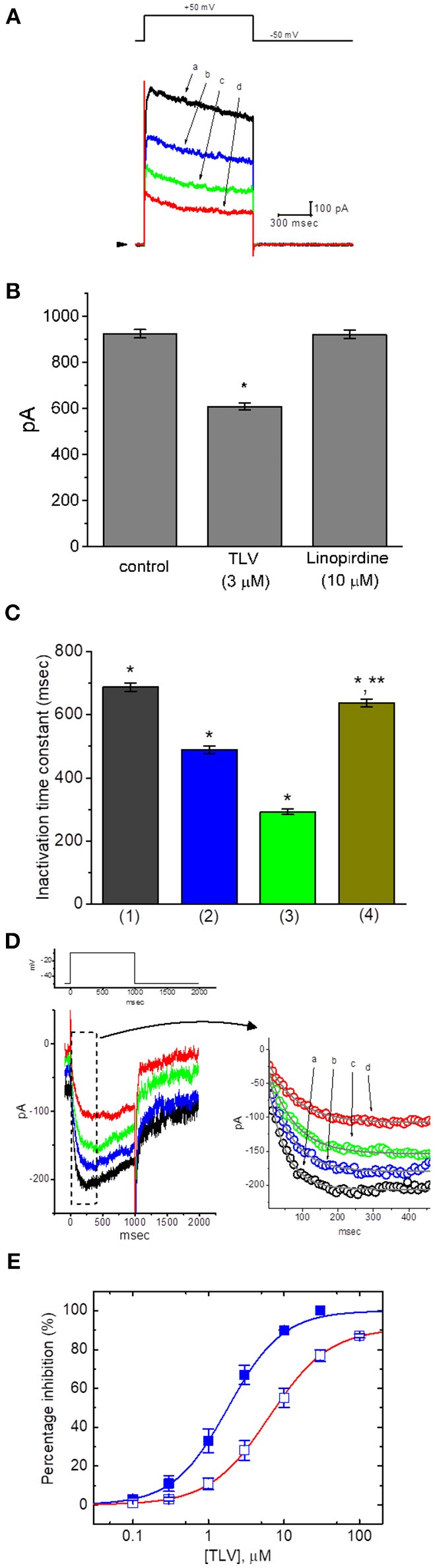
Effect of TLV on *I*_K(DR)_ and *I*_K(M)_ recorded from GH_3_ cells. The whole-cell current recordings for *I*_K(DR)_ or *I*_K(M)_ were conducted in cells bathed in Ca^2+^-free Tyrode's solution or in high-K^+^, Ca^2+^-free solution, respectively, and the recording pipettes used for both currents were filled with K^+^-containing solution. **(A)** Superimposed *I*_K(DR)_ traces elicited in response to membrane depolarization (indicated in the upper part). a: control; b: 3 μM TLV; c: 10 μM TLV; d: 30 μM TLV. Arrowhead indicates the zero current level. **(B)** Bar graph showing the effect of TLV (3 μM) or linopirdine (10 μM) on *I*_K(DR)_ amplitude in GH_3_ cells (mean ± SEM; *n* = 9–12 for each bar). *Significantly different from control (*P* < 0.05). **(C)** Bar graph showing the effect of TLV on inactivation time constant of *I*_K(DR)_ elicited by membrane depolarization (mean ± SEM; *n* = 9–12 for each bar). 1: control; 2: 3 μM TLV; 3: 10 μM TLV; 4: washout of 10 μM TLV. *Significantly different from control (*P* < 0.05) and **significantly different from TLV (10 μM) group (*P* < 0.05). **(D)** Superimposed *I*_K(M)_ by membrane depolarization (indicated in the upper part). The upper part in **(C)** indicates the voltage protocol applied, and the right part in **(C)** is an expanded record from dashed box indicating the TLV effect on *I*_K(M)_ trajectory in the absence or presence of different TLV concentrations. a: control; b: 0.3 μM TLV, c: 1 μM TLV; d: 3 μM TLV. Each current trajectory was least-squares fitted by a single exponential (indicated by the smooth line) with the activation time constant of 49.1 ms (a; in the absence of TLV), 63.3 ms (b. 0.3 μM TLV), 72.8 ms (c, 1 μM TLV), and 80.7 ms (d, 3 μM TLV). Notably, the data points (open circles) were reduced by a factor of 10 for clarity. **(E)** Concentration-dependent relationships of TLV on *I*_K(DR)_ (□) and *I*_K(M)_ (■) amplitude in GH_3_ cells. The relation between the percentage inhibition of *I*_K(DR)_ or *I*_K(M)_ and the TLV concentration is illustrated. Smooth curves are fits to a modified Hill function as described in section Materials and Methods. The values of IC_50_ required for inhibition of *I*_K(DR)_ and *I*_K(M)_ inherently in these cells are 6.42 and 1.91 μM, respectively, while the values for maximal suppression of those currents (i.e., E_max_) are 91 and 100%, respectively.

In the first set of whole-cell experiments, we tested whether TLV had any possible perturbations on *I*_K(DR)_ in GH_3_ cells. Cells were bathed in Ca^2+^-free Tyrode's solution and the recording pipette was filled with K^+^-containing solution, the composition of which was described above. Once whole-cell mode was established, the examined cells were held at−50 mV, and the depolarizing pulse to +50 mV with a duration of 1 s was then delivered to them. Consistent with previous observations (Wang et al., 2008; So et al., 2017), the *I*_K(DR)_ in response to such pulse protocol was drastically evoked in these cells. Unexpectedly, as cells were exposed to different concentrations of TLV, the *I*_K(DR)_ elicited by membrane depolarization in these cells was progressively decreased (Figure 1A). For example, TLV at a concentration of 3 μM decreased *I*_K(DR)_ amplitude to 608 ± 16 pA from a control value of 924 ± 18 pA (*n* = 12, *P* < 0.05). After washout of this compound, current amplitude returned to 917 ± 18 pA (*n* = 9, *P* < 0.05). Figure 1B illustrates the effect of TLV (3 μM) or linopirdine (10 μM) on *I*_K(DR)_ amplitude. Linopirdine is an inhibitor of M-type K^+^ currents (*I*_K(M)_). Moreover, addition of TLV (10 μM) was found to shorten the inactivation time constant of *I*_K(DR)_ trajectory fitted to a monoexponential decay significantly from 687 ± 13 to 293 ± 9 ms (*n* = 12, *P* < 0.05); and, washout of the agent, time constant returned to 638 ± 11 ms (*n* = 9, *P* < 0.05) (Figure 1C). The cell diameter between the absence and presence of TLV was not noted to differ significantly (32 ± 3 μm [in the control] vs. 31 ± 4 μm [in the presence of 10 μM TLV], *n* = 12, *P* > 0.05). In continued presence of 10 μM TLV, we did not observe that subsequent application of vasopressin (1 μM) produced any measurable effect on its suppression of *I*_K(DR)_. However, nonactin (10 μM), a K^+^ ionophore (Wu et al., 2012c), could reverse TLV-mediated suppression of *I*_K(DR)_ amplitude.

### Effect of TLV on I_K(M)_ in GH_3_ Cells

In the next set of experiments, we further explored the effect of TLV on other types of K^+^ currents (e.g., *I*_K(M)_) inherently in these cells. We immersed the cells in high-K^+^, Ca^2+^-free solution and, during each recording, then filled the electrode with K^+^-containing solution. There are several reasons why we used high-K^+^, Ca^2+^-free solution for measurement of *I*_K(M)_. First, removal of external Ca^2+^ in the bathing solution is to preclude the possible contamination of Ca^2+^-activated K^+^ currents in GH_3_ cells. Second, the amplitude of *I*_K(M)_ is relatively small as compared with that of *I*_K(DR)_; however, the activation of *I*_K(M)_ occurs near the level of resting membrane potential. Third, during cell exposure to high-K^+^, Ca^2+^-free solution at which the reversal potential is approximately zero, the potential applied would be near the resting membrane potential of the cells; consequently, amplified *I*_K(M)_ could be readily elicited in response to long-lasting depolarizing steps (Sankaranarayanan and Simasko, 1996). As illustrated in Figure 1D, when the depolarizing pulse from −50 to −10 mV was applied to the cell, a unique type of K^+^ inward current with the slowly activating and deactivating properties was readily evoked. This K^+^ current evoked in response to long-lasting membrane depolarization was subject to suppression by application of either linopirdine (10 μM) or pioglitazone (10 μM) and it has been hence identified as an *I*_K(M)_ (Sankaranarayanan and Simasko, 1996; Brown and Yu, 2000; Wu et al., 2012a; Hsu et al., 2014; Chen et al., 2018). Of particular interest, once whole-cell mode was established, as we exposed GH_3_ cells to different concentrations of TLV, the *I*_K(M)_ evoked by membrane depolarization from −50 to −10 mV progressively became diminished (Figure 1D). For example, there was a marked reduction of current amplitude from 179 ± 10 to 94 ± 8 pA (*n* = 11, *P* < 0.05) during cell exposure to 3 μM TLV. Moreover, as cells were exposed to 3 μM TLV, the estimated activation time constant of *I*_K(M)_ in response to long membrane depolarization to −10 mV from a holding potential of −50 mV was significantly elevated to 81 ± 7 ms (*n* = 11) from a control of 49 ± 6 ms (*n* = 11, *P* < 0.05). After washout of the drug, current amplitude returned to 171 ± 9 pA (*n* = 8).

### Concentration-Dependent Effects of TLV on I_K(DR)_ and I_K(M)_ in GH_3_ Cells

The suppressive effects of TLV at the different concentrations, in the range of 0.1–100 μM, on *I*_K(DR)_ and *I*_K(M)_ amplitudes were further examined and compared. As depicted in Figure 1E, on the basis of a modified Hill function, the IC_50_ values of this compound required for the inhibitory effect of this drug on *I*_K(DR)_ and *I*_K(M)_ measured at the end of depolarizing pulse were calculated to be 6.42 and 1.91 μM, respectively, while those for maximal inhibition of these two currents were 91 and 100%, respectively. For example, TLV at a concentration of 10 μM suppressed *I*_K(M)_ amplitude by 90%, while it at the same concentration suppressed *I*_K(DR)_ amplitude only by 55%. The results would indicate, therefore, that the inhibitory effect of TLV observed in GH_3_ cells was dependent on the specific types of K_V_ channels present.

### Effect of TLV on the Steady-State Activation Curve of I_K(M)_ Taken From GH_3_ Cells

**Figure 2:**
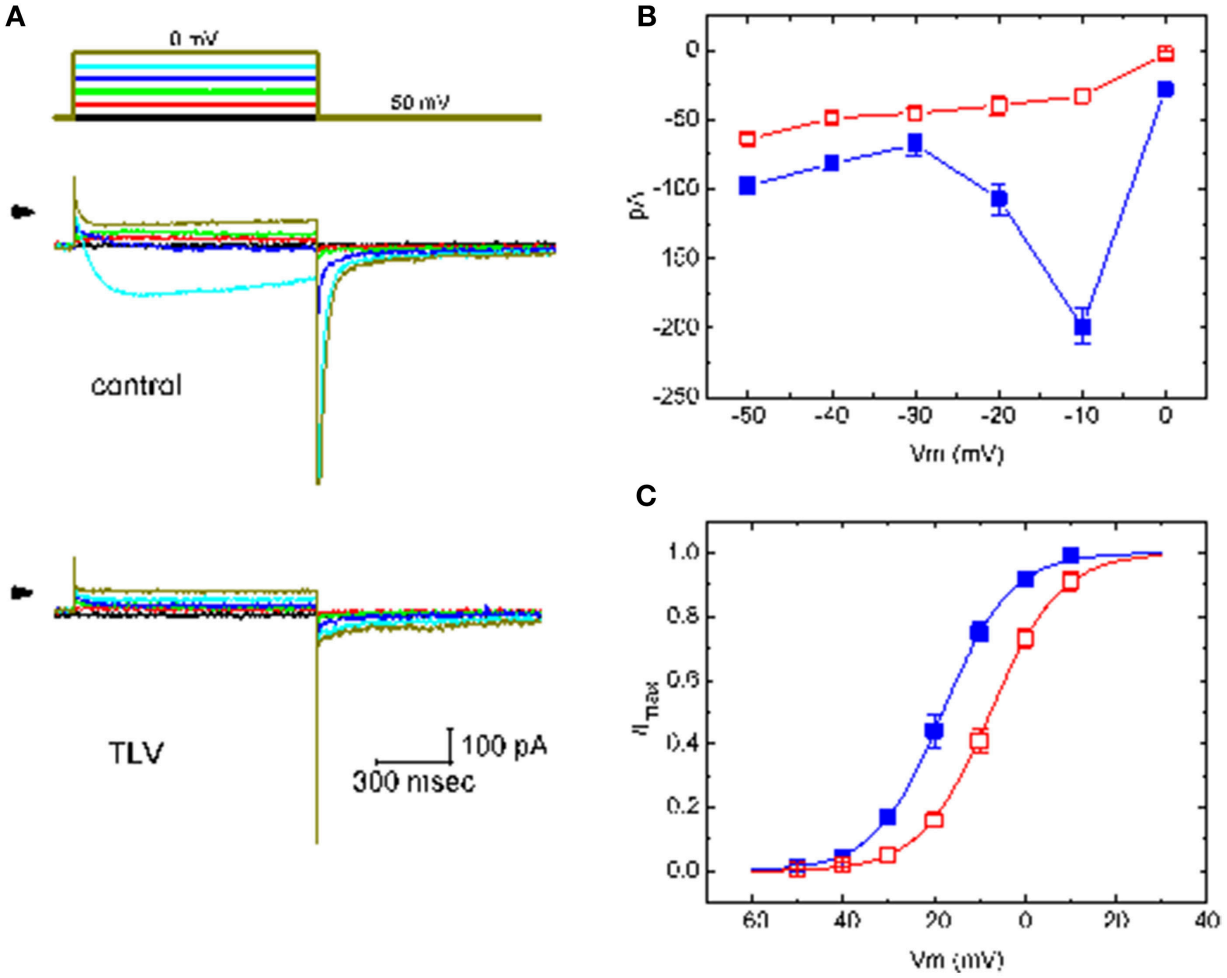
Effect of TLV on averaged *I-V* relationship of *I*_K(M)_ taken from GH_3_ cells. The whole-cell experiments on *I*_K(M)_ were conducted with high-K^+^, Ca^2+^-free solution and the cell was depolarized from −50 mV to various potentials ranging from −50 to 0 mV in 10-mV increments. **(A)** Superimposed *I*_K(M)_ traces elicited by various pulses (indicated in the uppermost part), which were obtained in the absence (upper) or presence (lower) of 10 μM TLV. Arrowhead in each panel indicates zero current level. **(B)** The relationship between *I*_K(M)_ amplitude and membrane potential obtained with or without addition of 10 μM TLV (mean ± SEM; *n* = 11 for each point). **(C)** The activation curve of *I*_K(M)_ in the absence and presence of 10 μM TLV (mean ± SEM; *n* = 9 for each point). The smooth curves were fitted by a Boltzmann function described in section Materials and Methods. In **(B,C)**, ■ is the control, and ? was obtained during the exposure to 10 μM TLV.

The effect of TLV on *I*_K(M)_ elicited by different pulses from a holding potential of −50 mV was also investigated. We conducted these experiments in the cells which were immersed in high-K^+^, Ca^2+^-free solution, and the depolarizing pulses from −50 mV to various potentials were delivered to the cells. The *I-V* relationship for inhibitory effect of TLV (10 μM) on *I*_K(M)_ amplitude is illustrated in Figures 2A,B. As cells were exposed to 10 μM TLV, the whole-cell *I*_K(M)_ conductance measured at the voltage ranging between −30 and −10 mV was evidently reduced to 0.63 ± 0.04 nS (*n* = 11) from a control value of 6.57 ± 0.11 nS (*n* = 11, *P* < 0.05). The steady-state activation curve of *I*_K(M)_ in the absence or presence of TLV was further analyzed. The curves obtained with or without the addition of TLV were plotted against the test potential and satisfactorily fit to the Boltzmann equation as described under Materials and Methods (Figure 2C). In control (i.e., in the absence of TLV), V_1/2_ = −18.1 ± 1.2 mV and *q* = 3.36 ± 0.08 *e* (*n* = 11), whereas during the exposure to TLV (10 μM), V_1/2_ = −7.6 ± 1.1 mV and *q* = 3.29 ± 0.08 *e* (*n* = 9). As such, it is evident from the results that the presence of TLV not only produced a considerable reduction in *I*_K(M)_ amplitude or conductance, but it also significantly shifted the steady-state activation curve of the current to a rightward direction by approximately 10 mV. The data from these analyses showed that the translocation of around 3 *e* across the electric field is responsible for the voltage dependence of *I*_K(M)_ in GH_3_ cells; however, minimal change in the estimated q value of the curve was detected during the exposure to TLV. Therefore, the voltage dependence of *I*_K(M)_ in these cells was virtually changed in the presence of TLV, although the movement through the electrical field in response to changes of membrane potential did not differ.

### Effect of TLV on *Erg*-Mediated K^+^ Current (I_K(erg)_) in GH_3_ Cells

**Figure 3:**
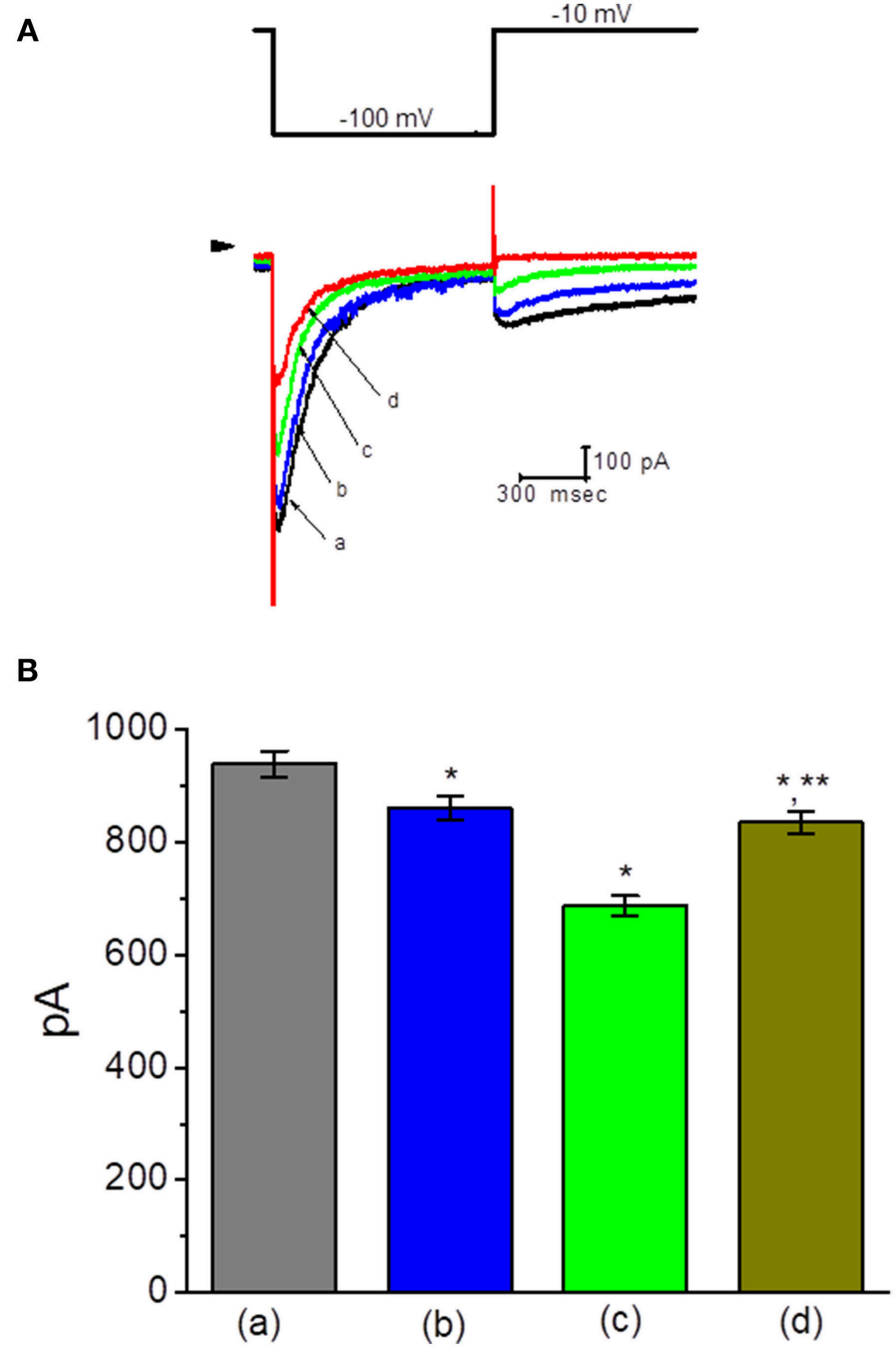
Effect of TLV on *erg*-mediated K^+^ current (*I*_K(erg)_) in GH_3_ cells. In this set of experiments, cells were bathed in high-K^+^, Ca^2+^-free solution, and the examined cell was held at −10 mV and the hyperpolarizing pulse to −100 with a duration of 1 s was delivered. **(A)** Superimposed *I*_K(erg)_ traces obtained in the control (a) and during cell exposure to 10 μM TLV (b), 30 μM TLV (c), and 100 μM TLV (d). Arrowhead indicates the zero current level, and the upper part is the voltage protocol applied. **(B)** Summary of the data showing effects of TLV and TLV plus PD-118057 on *I*_K(erg)_ amplitude in GH_3_ cells (mean ± SEM; *n* = 9 for each bar). a: control; b: 10 μM TLV; c: 30 μM TLV; d: 30 μM TLV plus 10 μM PD-118057. *Significantly different from control (*P* < 0.05) and **significantly different from TLV (30 μM) alone group (*P* < 0.05).

Another type of K^+^ current (i.e., *I*_K(erg)_) functionally expressed in these cells (Wu et al., 2012b) was also further examined. To measure *I*_K(erg)_, cells were bathed in high-K^+^, Ca^2+^-free solution and the pipette was filled with K^+^-containing solution, because under such external conditions, *I*_K(erg)_ could be amplified. However, upon membrane depolarization, K^+^ currents would be overlapped with different components of K^+^ currents including *I*_K(M)_, A-type K^+^ current (*I*_K(A)_), and *I*_K(DR)_. Under experimental conditions, as the whole-cell configuration was established, the membrane hyperpolarization from −10 to −100 with a duration of 1 s was applied to evoke *I*_K(erg)_. As shown in Figure 3A, by comparison with the experimental results from the effects on *I*_K(M)_ or *I*_K(DR)_, the presence of TLV suppressed *I*_K(erg)_ amplitude to a less magnitude. For example, TLV at a concentration of 10 μM slightly but significantly produced a decline in *I*_K(erg)_ amplitude only by 8%. However, in continued presence of TLV (30 μM), further addition of PD-118057 was able to reverse the inhibition of *I*_K(erg)_ caused by this compound, as evidenced by the elevation of *I*_K(erg)_ amplitude from 686 ± 18 to 834 ± 19 pA (*n* = 9, *P* < 0.05) (Figure 3B). PD-118057 was previously reported to enhance *I*_K(erg)_ amplitude (Zhou et al., 2005).

### Ability of TLV to Suppress the Activity of Large-Conductance Ca^2+^-Activated K^+^ (BK_Ca_) Channels Recorded From GH_3_ Cells

**Figure 4:**
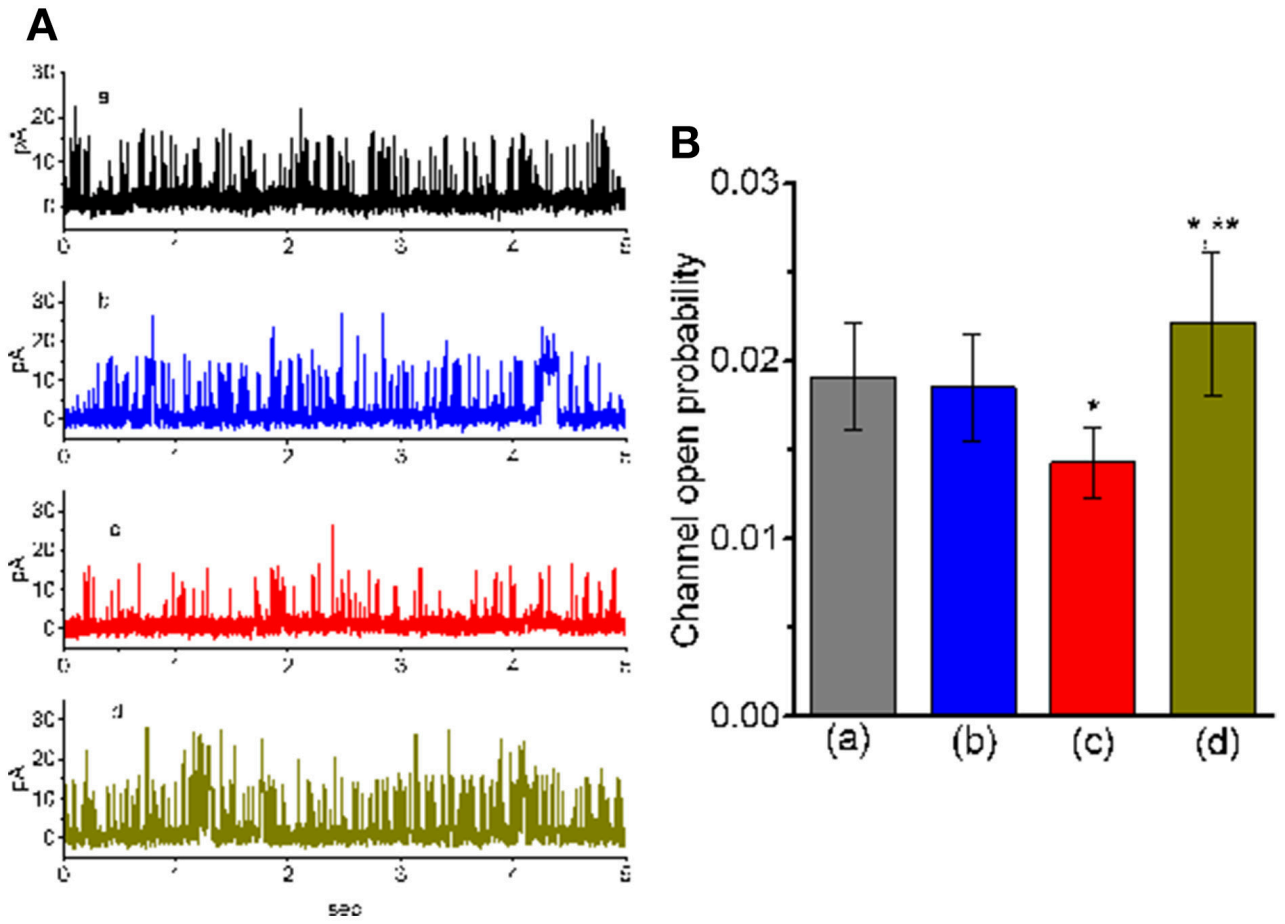
Effect of TLV and TLV plus cilostazol on BK_Ca_-channel activity in GH_3_ cells. These experiments were conducted with symmetrical K^+^ solution (145 mM). Under inside-out configuration, the potential was held at the level of +60 mV and bath medium contained 0.1 μM Ca^2+^. **(A)** Representative BK_Ca_-channel traces taken from a GH_3_ cell. The upper deflection indicates the opening event of the channel. a: control; b: 10 μM TLV; c: 30 μM TLV; d: 30 μM TLV plus 10 μM cilostazol. **(B)** Summary of the data showing effects of TLV and TLV plus cilostazol on the probability of BK_Ca_-channel openings (mean ± SEM; *n* = 12 for each bar). a: control; b: 10 μM TLV; c: 30 μM TLV; d: 30 μM TLV plus 10 μM cilostazol. *Significantly different from control or TLV (10 μM) alone group (*P* < 0.05) and **significantly different from TLV (30 μM) alone group (*P* < 0.05).

We next wanted to study if TLV can alter the activity of BK_Ca_ channels enriched in GH_3_ cells (Wu et al., 2004, 2017b; So et al., 2018). In these single-channel current recordings, cells were bathed in high-K^+^ solution containing 0.1 μM Ca^2+^, and each inside-out membrane patch was held at +60 mV. As depicted in Figure 4, when TLV at a concentration of 10 μM was applied to the cytosolic surface of the detached patch, the probability of BK_Ca_ channels that would be open was not changed significantly. However, addition of TLV (30 μM) was noted to reduce channel open probability significantly, along with no clear change in single-channel amplitude, as evidenced by a reduction of channel open probability from 0.0191 ± 0.003 to 0.0143 ± 0.002 (*n* = 12, *P* < 0.05). A prolongation of mean closed time of the channel was demonstrated in the presence of TLV (30 μM) (34 ± 8 ms [control] vs. 73 ± 13 ms [in the presence of 30 μM TLV], *n* = 9, *P* < 0.05); however, the mean open time did not differ between the presence and absence of 30 μM TLV. Moreover, subsequent addition of cilostazol (10 μM), still in the presence of TLV (30 μM), effectively reversed its suppressive effect on the probability of channel openings. Cilostazol was recognized as an activator of BK_Ca_ channels (Wu et al., 2004).

### Inability of TLV to Alter the Amplitude of Hyperpolarization-Activated Cation Current (I_h_) Recorded From GH_3_ Cells

**Figure 5:**
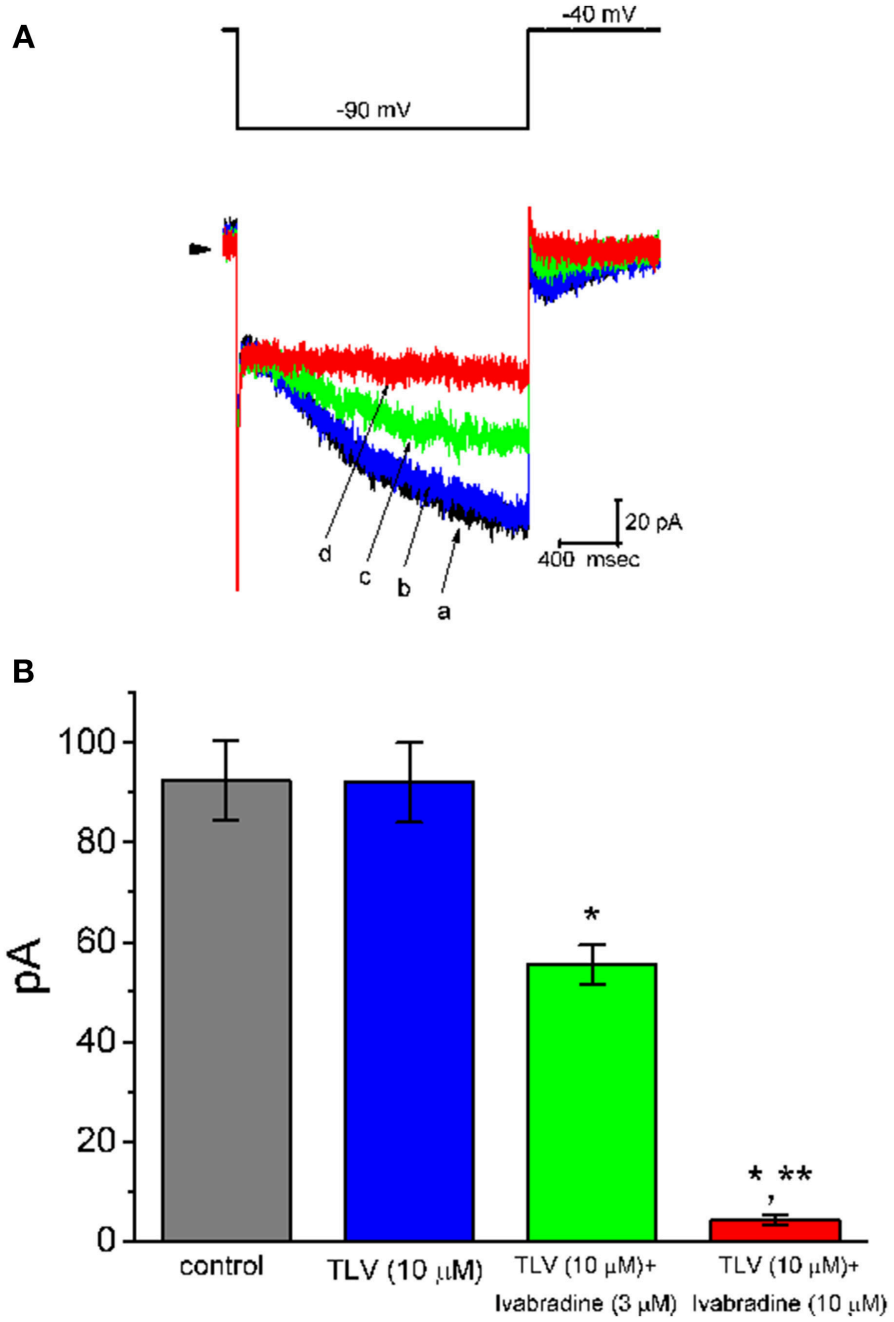
Effect of TLV and TLV plus ivabradine on hyperpolarization-activated cation current (*I*_h_) expressed in GH_3_ cells. In these whole-cell current recordings, cells were bathed in Ca^2+^-free Tyrode's solution. In each experiment, a long-step hyperpolarizing pulse from −40 to −100 mV with a duration of 2 s was delivered to the cell. **(A)** Superimposed *I*_h_ traces elicited by membrane hyperpolarization (indicated in the upper part). a: control; b: 10 μM TLV; c: 10 μM TLV plus 3 μM ivabradine; d: 10 μM TLV plus 10 μM ivabradine. **(B)** Summary of the data showing effects of TLV and TLV plus ivabradine on *I*_h_ amplitude in GH_3_ cells (mean ± SEM; *n* = 9 for each bar). The *I*_h_ amplitude was taken from the difference in current amplitude measured at the beginning and end of hyperpolarizing pulse. *Significantly different from control or TLV (10 μM) alone group (*P* < 0.05), and **significantly different from TLV (10 μM) plus ivabradine (3 μM) group (*P* < 0.05).

The next set of experiments was further conducted to assess if TLV could perturb the amplitude of *I*_h_ in these cells. Cells were bathed in Ca^2+^-free Tyrode's solution containing 1 μM tetrodotoxin, and the recording electrode was filled with K^+^-containing solution. When the holding potential was clamped at −40 mV and the hyperpolarizing pulse to −100 mV with a duration of 2 s was applied, the *I*_h_ elicited under this pulse protocol was readily observed as reported previously (Liu et al., 2009). As illustrated in Figure 5, the *I*_h_ amplitude elicited in response to such long-lasting membrane hyperpolarization was little changed during cell exposure to 30 μM TLV. However, further addition of ivabradine (10 μM), but still in the presence of 30 μM TLV, was effective at suppressing the amplitude of *I*_h_, along with a considerable slowing in the activation time course of the current. Ivabradine was previously recognized as an inhibitor of *I*_h_ (Romanelli et al., 2016). Therefore, distinguishable TLV effects on *I*_K(DR)_ or *I*_K(M)_ as described above, the *I*_h_ amplitude in response to membrane hyperpolarization in these cells was resistant to be changed by this drug.

### Effect of TLV on I_K(M)_ in GH_3_ Cells Preincubated With Vasopressin

**Figure 6:**
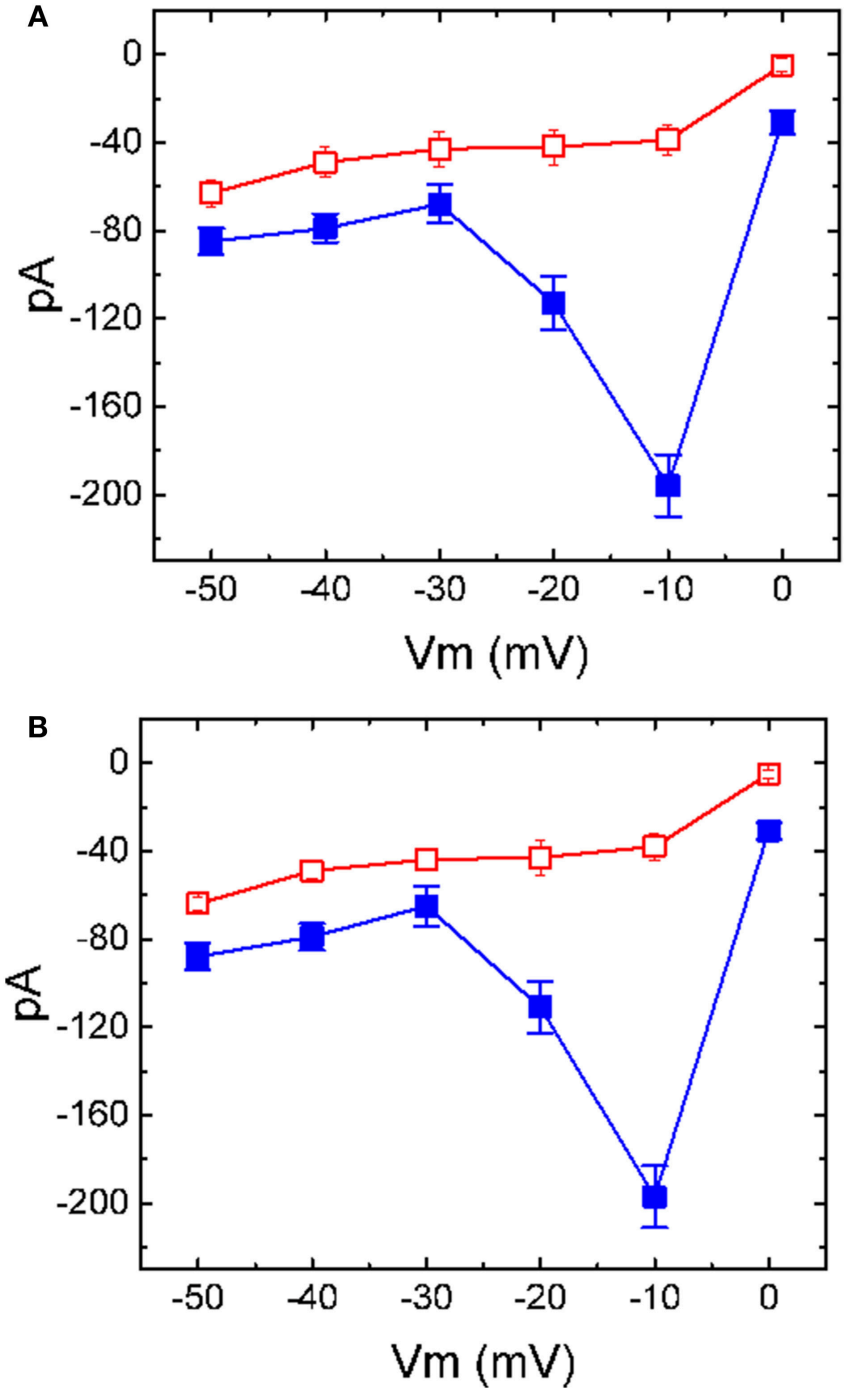
Inhibitory effect of TLV on *I-V* relationship of *I*_K(M)_ in GH_3_ cells preincubated with or without vasopressin. In **(A)**, the data were obtained from control cells (i.e., in the absence of vasopressin treatment), while in **(B)**, those were from cells preincubated with vasopressin (1 μM) for 6 h. In each experiment, cells were bathed in high-K^+^, Ca^2+^-free solution, the examined cell was held at −50 mV and the voltage pulse ranging between −50 and 0 mV with 10-mV increments was applied. Each point represents the mean ± SEM (*n* = 8).■: control; □: in the presence of 10 μM TLV.

It has been shown that the effect of TLV was predominantly associated with its blockade of vasopressin receptor (Ghali et al., 2009; Izumi et al., 2014; Clark et al., 2017; Berardi et al., 2018; Matsukawa et al., 2018). Vasopressin *per se* tends to regulate the activities of various ion channels (Nakajima et al., 1996). Moreover, the binding of vasopressin receptor by vasopressin might influence functional activities of anterior pituitary cells including GH_3_ cells (Liu and Ben-Jonathan, 1994; Izumi et al., 2014). For these reasons, the effect of TLV on *I*_K(M)_ was further evaluated in cells treated with vasopressin (1 μM) for 6 h. Of note, as compared with those effects in control cells, in GH_3_ cells preincubated with vasopressin (1 μM) for 6 h, inhibitory effect of TLV on averaged *I-V* relationship of *I*_K(M)_ was little altered (Figure 6). For example, in cells pretreated with vasopressin (1 μM), addition of TLV (10 μM) evidently decreased *I*_K(M)_ amplitude at the level of −10 mV from 197 ± 14 to 39 ± 8 pA (*n* = 7, *P* < 0.05). These results would suggest, therefore, that the observed effect of TLV on the inhibition of *I*_K(M)_ is not solely explained by its blockade of vasopressin receptors possibly expressed in GH_3_ cells.

### Effect of TLV and TLV Plus Flupirtine on Spontaneous Action Potentials (APs) in GH_3_ Cells

**Figure 7:**
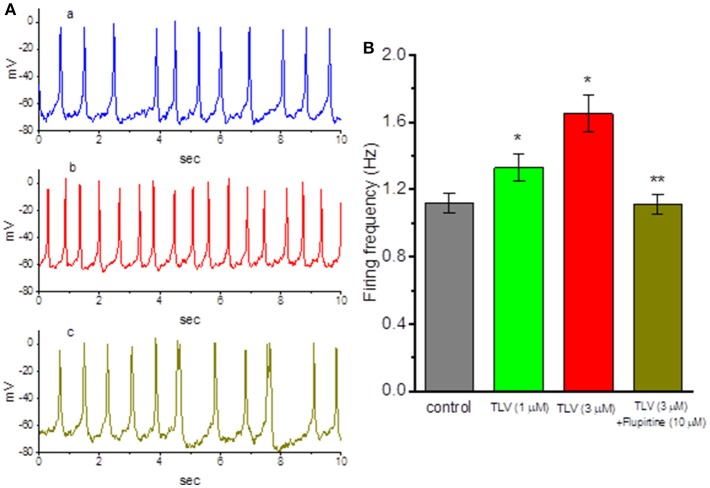
Effects of TLV and TLV plus flupirtine on spontaneous action potentials (APs) in GH_3_ cells. In these experiments conducted in whole-cell current-clamp voltage recordings, cells were bathed in normal Tyrode's solution containing 1.8 mM CaCl_2_ and the recording pipette was filled with K^+^-containing solution. **(A)** Potential trace labeled a is control, and those labeled b and c were obtained after addition of 3 μM TLV and 3 μM TLV plus 10 μM flupirtine, respectively. **(B)** Summary of the data showing effects of TLV and TLV plus flupirtine on the firing frequency of spontaneous APs (mean ± SEM; *n* = 12 for each bar). *Significantly different from control (*P* < 0.05) and **significantly from TLV (3 μM) alone group (*P* < 0.05).

In another set of experiments, we wanted to determine whether TLV affects spontaneous APs in these cells. Cells were bathed in normal Tyrode's solution and the current-clamp voltage recordings were made to measure the occurrence of spontaneous APs. Of note, as cells were exposed to TLV, the firing of spontaneous APs was progressively elevated (Figure 7). For example, TLV (3 μM) increased the firing frequency from 1.12 ± 0.05 to 1.65 ± 0.11 Hz (*n* = 12, *P* < 0.05). As we exposed GH_3_ cells to TLV (3 μM), the resting potential in these cells also became significantly depolarized from −68.1 ± 0.9 to −59.2 ± 1.2 mV (*n* = 12, *P* < 0.05). Moreover, still in the presence of TLV, further application of flupirtine (10 μM), a known activator of *I*_K(M)_ (Wu et al., 2012a), was able to reverse TLV-stimulated increase of AP firing, as evidenced by a significant reduction of firing frequency to 1.11 ± 0.06 Hz (*n* = 12, *P* < 0.05). Therefore, the results suggest that TLV-mediated changes of membrane potential in GH_3_ cells ascribe largely from its inhibition of *I*_K(M)_. Since *I*_K(M)_ does not reach at its maximum, the increase of *I*_K(M)_ caused by flupirtine might be responsible for its attenuation in TLV-mediated increase of the firing rate.

### Effect of TLV on I_K(DR)_ in Madin-Darby Canine Kidney (MDCK) Cells

**Figure 8:**
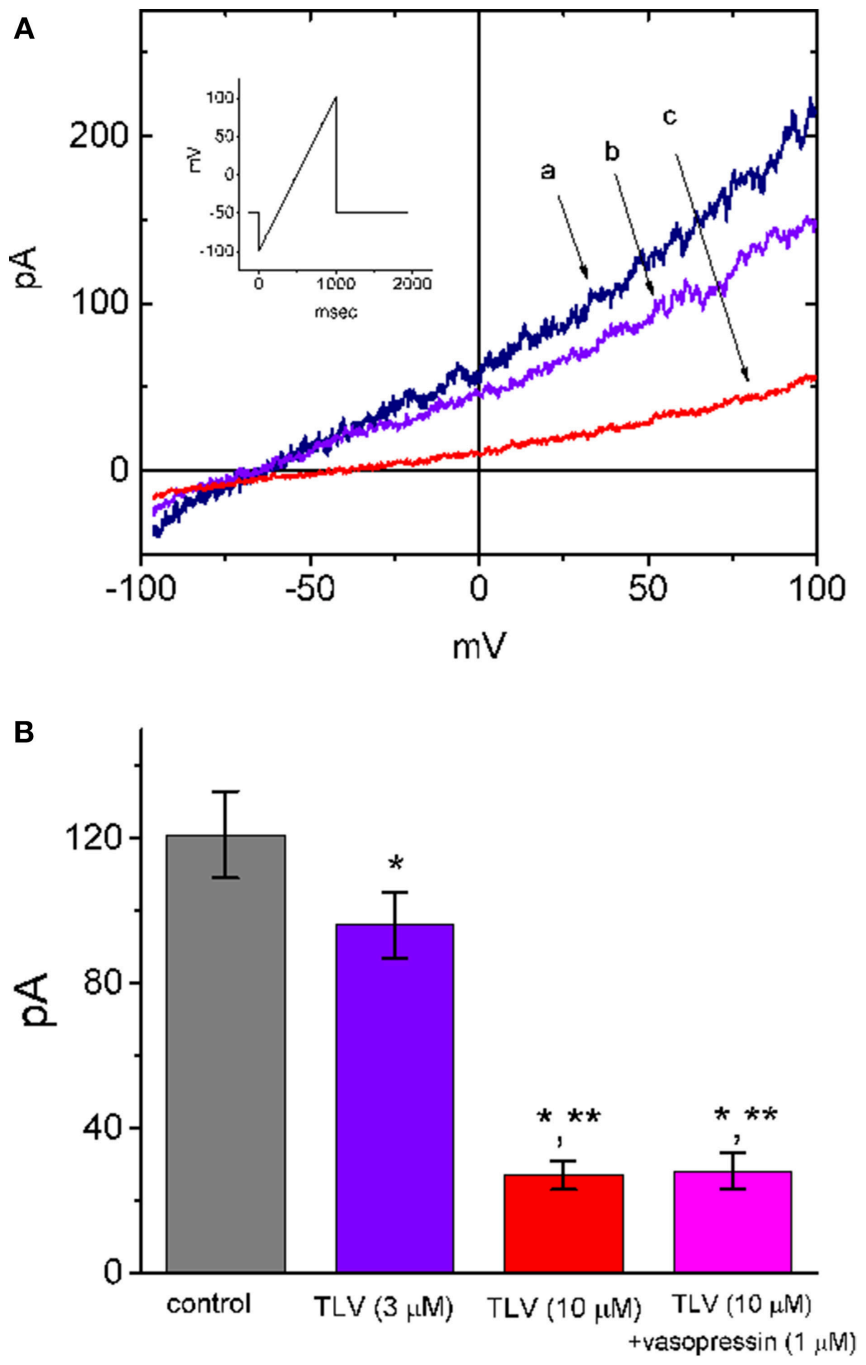
Effect of TLV on *I*_K(DR)_ present in MDCK cells. In these experiments, cells were bathed in Ca^2+^-free Tyrode's solution and the electrode was filled with K^+^-containing solution. As whole-cell configuration was established, we held the examined cell at the level of −50 mV and the ramp pulse from −100 to +100 mV with a duration of 1 s was then delivered to it. **(A)** Representative *I*_K(DR)_ traces in response ramp pulse (indicated in Inset). a: control; b: 3 μM TLV; c: 10 μM TLV. **(B)** Summary of the data showing effects of TLV, TLV plus vasopressin (1 μM) and TLV plus nonactin (10 μM) on *I*_K_ amplitude in MDCK cells (mean ± SEM; *n* = 9 for each bar). Current amplitude was measured at the level of +50 mV. *Significantly different from control (*P* < 0.05) and **significantly different from TLV (3 μM) alone (*P* < 0.05).

Because TLV is able to exert significant function on renal epithelial cells (Tamma et al., 2017), another set of experiments was conducted in MDCK cells to determine whether this drug has any effect on *I*_K(DR)_. The MDCK cell line has been a useful model for the investigations on functional activities in renal tubular cells (Lang and Paulmichi, 1995; Jan et al., 1999; David et al., 2013; Wu et al., 2015; Tamma et al., 2017). To evoke *I*_K(DR)_, cells were bathed in Ca^2+^-free Tyrode's solution, the potential was held at −50 mV, and the ramp pulse from −100 to +100 mV with a duration of 1 s was thereafter delivered. Notably, addition of TLV suppressed *I*_K(DR)_ amplitude effectively in these cells (Figure 8). For example, at the level of +50 mV, TLV (10 μM) could significantly decrease *I*_K(DR)_ amplitude from 121 ± 12 to 27 ± 4 pA (*n* = 9, *P* < 0.05). However, subsequent addition of vasopressin (1 μM), still in the presence of 10 μM TLV, failed to counteract the *I*_K(DR)_ suppression by TLV.

## Discussion

In the present study, we revealed that TLV effectively and differentially suppressed the amplitude of *I*_K(DR)_ and *I*_K(M)_ in a concentration- and time-dependent fashion in pituitary GH_3_ cells. This drug slightly inhibited *I*_K(erg)_ amplitude and BK_Ca_-channel activity, while it had little or no effect on *I*_h_. Under whole-cell voltage recordings, cell exposure to this compound was able to depolarize the membrane potential and to cause an increase in firing frequency of spontaneous APs. In MDCK cells, the presence of TLV was also effective at suppressing *I*_K(DR)_ evoked by ramp pulse.

The IC_50_ values of TLV needed to suppress *I*_K(DR)_ and *I*_K(M)_ observed in GH_3_ cells were estimated to be 6.42 and 1.91 μM, respectively, indicating a 3.4-fold selectivity for its suppression of *I*_K(DR)_ vs. *I*_K(M)_. Additionally, our study revealed the ability of this compound to shift the steady-state activation curve of *I*_K(M)_ to less depolarized potential, despite no measurable change in the gating charge of the curve in its presence, indicating that its presence could suppress *I*_K(M)_ amplitude in a voltage-dependent fashion in these cells. Therefore, despite that the detailed ionic mechanisms of TLV remain to be resolved, changes in the amplitude of *I*_K(M)_ during its exposure were rapid over time and were sensitive to the preexistent resting potential, the concentration used, or both.

Vasopressin receptors have been previously reported to exist in anterior pituitary cells including GH_3_ cells (Liu and Ben-Jonathan, 1994; Izumi et al., 2014). TLV has the propensity to interfere with the actions of vasopressin as a neurotransmitter at vasopressin receptors inherently in GH_3_ cells. In our study, subsequent addition of neither vasopressin (1 μM) nor thyrotropin releasing hormone (1 μM), however, evidently reversed the suppression by TLV of *I*_K(DR)_ or *I*_K(M)_ seen in these cells. Moreover, in GH_3_ cells preincubated with vasopressin (1 μM), the inhibitory effect of TLV on these K^+^ currents remained unaffected. Vasopressin at a concentration of 1 μM was reported to activate vasopressin receptors maximally (Armstrong et al., 2013). Therefore, findings from the present results led us to propose that the ability of TLV on suppress the amplitude of *I*_K(DR)_, *I*_K(M)_ or both is most likely to be independent of the mechanisms linked to either the blockade of vasopressin V_2_ receptors (Ghali et al., 2009; Izumi et al., 2014; Clark et al., 2017; Berardi et al., 2018; Matsukawa et al., 2018) or the elevation in the level of intracellular inositol trisphosphate (Quintero et al., 2005).

The peak plasma concentration of TLV following single oral dose of TLV as 60-mg tablet was previously reported to reach about 720–1300 ng/ml (about 1.6–2.9 μM) (Shoaf et al., 2007). Therefore, besides the blockade of vasopressin receptor, the inhibitory effects of TLV on different types of K^+^ currents found in this study may occur within clinically therapeutic range, and they could be a potentially important mechanism through which the drug perturbs membrane excitability of endocrine or neuroendocrine cells, if similar *in vivo* findings occur. As such, it is tempting to anticipate that both such direct inhibition of multiple K^+^ currents and blockade of vasopressin V_2_ receptor by this drug or other structurally similar non-peptide compounds produce beneficial effects on patients with hyponatremia or syndrome of inappropriate antidiuretic hormone secretion.

The K_V_7.1-encoded current has been notably reported to be functionally expressed in polarized MDCK cells (Jespersen et al., 2004; David et al., 2013). The suppression of K^+^ currents by TLV in renal epithelial cells could virtually exert an alternative impact on the harness of vasopressin-induced water movement. Synergistic antagonism of vasopressin V_2_ receptor and inhibition of these K^+^ currents caused by TLV at the concentrations noted to be achievable in humans (Shoaf et al., 2007; Kato et al., 2016; Oguri et al., 2018a), may also potentially account for its actions on renal epithelial cells. Therefore, determining to what extent such inhibitory actions by TLV or other structurally similar compounds (Tabata et al., 2017) contribute to their therapeutic effectiveness in renal water excretion is worthy of being imperatively investigated, as they have been rampantly used in different types of electrolyte disorders such as hyponatremia (Izumi et al., 2014; Aylwin et al., 2015; Verbalis et al., 2016; Clark et al., 2017; Der-Nigoghossian et al., 2017; Dunlap et al., 2017; Felker et al., 2017; Konstam et al., 2017; Wu et al., 2017a; Berardi et al., 2018; Kogure et al., 2018; Matsukawa et al., 2018; Morris et al., 2018; Sigal et al., 2018; Vidic et al., 2019).

Tricyclic antidepressants such as imipramine have been demonstrated to suppress various types of K^+^ currents including *I*_K(DR)_ and *I*_K(M)_ (Casis et al., 2002; Quintero et al., 2005). As such, whether an alternative effect on depression or anxiety disorders by the blockers of vasopressin V_1B_ receptor as described previously (Iijima et al., 2014) is linked to their possible actions on multiple types of K^+^ currents remains to be essentially resolved. Alternatively, in addition to the improvement of hyponatremic condition due to water diuresis, TLV may directly ameliorate cognitive function through a mechanism linked to its effective suppression at multiple K^+^ currents, particularly at *I*_K(M)_, inherently in central neurons (Soiza and Talbot, 2011; Graziani et al., 2012; Ahluwalia et al., 2015; Verbalis et al., 2016; Der-Nigoghossian et al., 2017; Chen et al., 2018).

In light of the present study, despite the antagonistic effect on vasopressin V_2_ receptors (Ghali et al., 2009; Izumi et al., 2014; Clark et al., 2017; Berardi et al., 2018; Matsukawa et al., 2018), our results strongly suggest that the inhibitory effects of TLV on multiple ion channels, particularly on K^+^ currents, tend to be obligate mechanisms. Through ionic mechanisms presented herein, it or other structurally similar non-peptide compounds that can be preferentially used for oral intake, is able to influence the functional activities of endocrine or renal tubular cells, if similar findings occur *in vivo*. Our findings also highlight an important alternative aspect that needs to be taken into account, inasmuch as the aquaretic effects of TLV in different pathologic disorders including polycystic kidney disease, cirrhosis or heart failure, are evaluated (Ghali et al., 2009; Graziani et al., 2012; Mancinelli et al., 2016; Clark et al., 2017; Sweeney and Avner, 2017; (Torres et al., 2017; van Gastel and Torres, 2017; Wu et al., 2017a; Chebib et al., 2018; Edwards et al., 2018; Imamura and Kinugawa, 2018; Matsukawa et al., 2018; McEwan et al., 2018; Müller et al., 2018; Oguro et al., 2018b; Poch et al., 2018; Sen et al., 2018; Takimura et al., 2018).

## Author Contributions

T-LL contributed to writing and experiments. W-TC contributed to experiments and materials. C-HC contributed to materials preparation and S-NW was responsible for experiment design, data collection and manuscript writing.

### Conflict of Interest Statement

The authors declare that the research was conducted in the absence of any commercial or financial relationships that could be construed as a potential conflict of interest.

## Abbreviations

AP: action potential
BK_Ca_ channel: large-conductance Ca^2+^-activated K^+^ channel
*erg*: *ether-à-go-go*-related gene
*I-V*: current vs. voltage
*I*_h_: hyperpolarization-activated cation current
*I*_K(DR)_: delayed-rectifier K^+^ current
*I*_K(erg)_: *erg*-mediated K^+^ current
*I*_K(M)_: M-type K^+^ current
K_V_ channel: voltage-gated K^+^ channel
MDCK cell: Madin-Darby canine kidney cell
SEM: standard error of the mean
TLV: tolvaptan.
